# Teaching the moving body with a still pedagogy: body percussion and the coupling architecture of embodied learning in physical education

**DOI:** 10.3389/fspor.2026.1963569

**Published:** 2026-10-14

**Authors:** Francisco Javier Romero-Naranjo

**Affiliations:** Department of Innovation and Didactic Training, University of Alicante, Alicante, Spain

**Keywords:** body percussion, embodied learning, interpersonal entrainment, motor learning, physical education pedagogy, sensorimotor synchronization, transfer

## Abstract

Embodied learning is an influential orientation in physical education and sport pedagogy, but the field lacks agreement on which property of a movement practice does the educational work. Function-based frameworks locate it in the epistemic function that movement performs. This paper proposes one such function for rhythmic group practices: the distribution of influence over a shared timing structure. External entrainment is defined as unidirectional coupling to a source that performers cannot influence, and mutual entrainment as bidirectional coupling in which each participant is at once sound source and receiver. The core claim is that reducing the asymmetry of that distribution widens the range of coordinative solutions a group explores, improves transfer to untrained patterns and tempi, and speeds recovery after perturbation, at a cost in fidelity to the trained pattern. The proposed active ingredient is contingency rather than variability: under mutual coupling a participant's correction alters the target, which an irregular but unresponsive source does not. This contrast supplies the principal test and separates the proposal from contextual interference and constraints-led accounts. The Coupling Asymmetry Index is built from established measures: pairwise phase-correction gains, estimated as in published work on string quartets, are aggregated as the complement of weighted network reciprocity, which makes the index invariant to group size. A mean-field regime, in which participants adapt to the ensemble aggregate, is distinguished and shown to limit the group size at which reciprocity can be sustained. Body percussion is the exemplary case because it is the classroom practice in which coupling architecture is a free design variable. Two further claims follow. Current practice and evidence in body percussion are prescriptive despite an embodied justification, a claim stated as a testable hypothesis with a survey protocol. And this default is an institutional frame factor inherited from the material organization of the classroom rather than a property of any method. The paper derives boundary conditions, an observational framework for practitioners, ten falsifiable propositions and a five-item reporting standard.

## 1 Introduction

Embodied cognition holds that thinking and learning are constituted by the interplay between body, mind and environment rather than merely accompanied by it (1, 2). The orientation is influential in physical education, but it is broad enough to justify almost any bodily activity, and there is no agreement on which property of a practice does the educational work. Function-based frameworks distinguish the bodily engagement a task demands from the way that engagement is integrated with what is to be learned (3), so that what matters is the epistemic function the movement performs. This paper proposes such a function for school practices in which a group produces a shared rhythm.

The proposed function is the distribution of influence over the shared timing structure. In some arrangements the pulse comes from a source the performers cannot influence, such as a teacher, a metronome or a recording, and coupling is unidirectional. In others it is generated by the participants, each of whom is at once a sound source and a receiver, and coupling is bidirectional (4, 5). Because the two arrangements leave identifiable adaptation signatures in joint tapping (6–8), the distinction can be measured. The core claim is that reducing the asymmetry of this distribution, so that the group generates and repairs its own timing, should widen the range of coordinative solutions explored, improve transfer to untrained patterns and tempi, and speed recovery after perturbation, at a cost in fidelity to the trained pattern.

Body percussion, the use of the body as a sound source for educational purposes (9), is the exemplary case. In most classroom movement practices the sound source is external by necessity; in body percussion it is produced by the bodies that must coordinate, so coupling architecture becomes a free design variable. The argument extends to dance, drumming ensembles and any group practice that can produce its own pulse. Part of the field places body percussion within the sciences of physical activity and sport under the label neuromotricity and relates it to the motor content of the school subject (10), including fundamental movement skills, whose health benefits in children and adolescents are well documented (11,76).

The practice, however, does not follow its own premises. In the canonical session an adult produces the pulse or selects the recording that does; a pattern is demonstrated and imitated; departures are corrected; success is fidelity of reproduction. These are the features of what Biesta (12) calls strong education and Freire (13) banking education, and of the “stilling in” of a pre-existing design that Ingold (14) contrasts with education. The evidence base has a similar structure: a bibliometric analysis of 245 documents published between 2001 and 2022 found a literature dominated by foundational work and, among empirical studies, by quasi-experimental pretest-posttest designs (15).

The paper follows van der Kamp et al. (16), who examined how researchers of radical embodied cognitive science teach their science through the prescriptive pedagogy their research argues against. The same analysis is applied here to body percussion, with two additions: the mismatch is given a historical explanation, and it is given an operational definition that allows it to be measured and, if the analysis is wrong, disconfirmed. Two further framings are adopted. Boundary conditions are treated as seriously as mechanisms, and teaching is understood to be shaped by frame factors, the organizational and material features of the school setting that lie outside the teacher's control and limit which instructional processes are possible (17).

Section 2 reviews the literatures on which the argument rests. Section 3 examines the mismatch and states it as a testable hypothesis, and section 4 explains its historical origin. Section 5 defines the coupling variable, its measurement, mechanism and boundary conditions. Section 6 proposes an observational framework, and sections 7 and 8 set out the propositions and a reporting standard.

## 2 Theoretical background

### 2.1 Ecological accounts and their pedagogical entailments

Within the ecological approach, skill learning arises from the interaction of performer, environmental and task constraints rather than from the performer alone (18). Instruction and demonstration are further constraints without logical priority, which prompt search rather than specify a solution (19, 20). Learning is coming to inhabit a richer landscape of affordances (21), and the teacher's task is the education of attention (16, 22). Proponents hold that it is futile to identify a single idealized motor pattern to which all learners should aspire (23).

Two entailments follow for any practice that claims this warrant. Movement variability is functional rather than residual, because it is the medium in which adaptive solutions are found. And since attention is educated through exploratory activity, learners require some control over the conditions of practice, including their timing. The position is not universally accepted, and the argument does not depend on it. Dreyfus and Dreyfus (24) held that instruction and modelling play a genuine role by allowing experience to accumulate rather than by transmitting an ideal pattern. The claim here is compatible with that view: prescription is not illegitimate, but in body percussion it is an undeclared default, neither reported nor manipulated.

### 2.2 Entrainment and the architecture of coupling

Entrainment is spatiotemporal coordination arising from rhythmic responsiveness to a perceived rhythmic signal. Phillips-Silver et al. (5) distinguish self-entrainment, where the signal is one's own output; social entrainment, where it originates in another agent; mutual entrainment, where two agents adjust reciprocally; and collective entrainment, where reciprocal adjustment runs through a group. What they term social entrainment is called external entrainment here, because the property at issue is the unresponsiveness of the source rather than whether it is an agent: on that criterion a conductor and a metronome occupy the same position.

The distinction has empirical support. In joint finger tapping, when both members of a dyad hear each other their inter-tap intervals adjust in opposite directions from tap to tap, a signature absent when one partner follows an unresponsive computer, and the dyads synchronize with each other as well as with the regular computer although the human partner is less regular (6). The underlying error-correction processes, phase and period correction, are described in enough detail to be estimated in applied settings (7, 8). Preschool children synchronize their drumming more accurately with a live partner than with a machine (25).

Brown (4) draws the consequence for practices in which performers are themselves the sound source. He calls the standard external and individualist account of the origins of human rhythm the dancing cockatoo model, after a cockatoo that entrains to recordings of pop music, and notes that under external entrainment those who move can only follow. He proposes instead a mutual model in which participants generate the beat, for instance through body percussion, and in which the functional advantage of entrainment is cooperative action.

A third arrangement matters as groups grow. In mean-field models of coupled oscillators, each element adjusts to the aggregate rhythm of the population rather than to identifiable partners (26, 27). In a large ensemble this is the more plausible regime, since beyond the number of performers a participant can distinguish by ear, what is heard is the group sound. Its consequences for measurement and for group size are set out in sections 5.1 and 5.4. That the direction of adaptation can itself be manipulated has been shown in engineered form: Moens and Leman (28) describe a music player that adapts its tempo to a walker's cadence, and distinguish the alignment strategies of finding the beat, keeping the beat and being in phase.

### 2.3 Variability, degeneracy and joint action

The mechanism of section 5.3 draws on two further literatures. The first concerns variability. Degeneracy, the capacity of structurally different coordinative structures to achieve the same function, has been proposed as the basis of the functional role of coordination variability (29). Motor learning research has shown independently that conditions which increase difficulty during acquisition, notably high contextual interference, can depress acquisition performance while improving retention and transfer (30, 31).

The second is joint action. Coordinating with others involves representing a shared task, predicting others' actions and adjusting one's own (32). In a group that produces its own pulse, each performer executes, predicts and corrects with respect to several others at once, which is a different task from tracking an unresponsive reference. The distinction concerns task structure, not the freedom granted to learners, a point developed in section 5.5.

## 3 The mismatch in body percussion

### 3.1 Coupling architecture in current practice

Research on the music teacher's body describes instructional gestures that explain an activity, guiding gestures that signal entries and endings, representational gestures, physical modelling and touch to correct posture (33, 34). Shifting instruction from language to the body can increase accessibility for young learners, for pupils with limited command of the language of schooling and for pupils with disabilities (33, 35).

These resources are pedagogically valuable and structurally prescriptive. Physical modelling has been criticized as teacher-directed and as restricting creativity (36), and although the teacher may adjust gestures in response to learners (37), the default direction of adjustment runs from teacher to class. In body percussion this asymmetry is compounded, because the teacher is usually both the model and the source of the pulse. The standard session therefore implements unidirectional coupling: the class adapts to a source that does not adapt to the class.

### 3.2 What the evidence base measures

Intervention research in body percussion and related rhythmic programmes has addressed cognitive and motor outcomes in school populations and older adults (38–40). Two features of this corpus matter here. First, the designs measure product rather than process: a pretest-posttest comparison shows that a difference occurred, not what participants did or how variable their solutions were. Second, the pedagogical structure of the intervention is rarely reported. Descriptions specify duration, frequency, population and content, but not who set the tempo, whether participants could alter it, whether alternative solutions were accepted or how error was defined. Under an ecological account these are the manipulated variables, so studies coded as delivering the same intervention may differ on the decisive dimension. A parallel argument has been made for second language phonology, where a hypothesis-generating coding of eleven intervention studies found that whether the movement was temporally aligned with a linguistic event, rather than the use of body movement as such, was the more consistent correlate of outcome (41).

A few studies compare active conditions. A randomized controlled trial with 475 students aged 11–12 with attention deficit hyperactivity disorder ran five arms over eight months: a dual-task rhythmic protocol, traditional physical education, choreographic body percussion, Orff music education and a passive control; a companion study adds a school orchestra (42, 43). A pilot study compared a rhythmic physical activity programme with standard physical education in children aged 6–11 (39), and a mixed-methods study with older adults at risk of dementia contrasted improvisational creative dance with choreographed body percussion (40). The field has thus begun to compare types of rhythmic practice rather than rhythmic practice against its absence, which is the level at which this framework operates.

These conditions share a property that has not, to my knowledge, been noticed. Table 1 arrays them by cognitive load and by estimated coupling asymmetry, expressed as the Coupling Asymmetry Index (CAI) defined in section 5. The conditions differ in load, yet in each the pulse appears to be set by a source the participants do not influence: a conductor, a recording or a memorized sequence performed to an external tempo. The qualification is necessary because the reports rarely say. The rhythmic programme of Vazou et al. (39) moved to the beat of music while the children created their own patterns, which shows that freedom over the pattern and control of the pulse are separate variables; for the dual-task arm of the trial the reader cannot tell who produced the beat. This reflects not carelessness but the fact that the field has not yet treated the variable as one requiring description, and section 3.3 applies that standard to the author's own trial first.

**Table 1:** Comparison of conditions typically used in trials of rhythmic movement interventions, arrayed by cognitive load and estimated coupling asymmetry.

Condition	Typical pulse source	Estimated CAI	Cognitive load
Passive control	None	Not applicable	Baseline
Traditional physical education	Teacher instruction; game structure	High	Low
School orchestra	Conductor and score	Very high	Moderate
Orff instrumental work	Teacher, score, recording	High	Moderate
Choreography-based body percussion	Memorized sequence performed to an external tempo	High	Moderate, decreasing with automation
Dual-task rhythmic protocols	Not reported	Unspecified	High

CAI, Coupling Asymmetry Index, defined formally in Section 5.2. Estimates are the author's inferences from published descriptions, not measured values; the inability to do better is the point of Section 8.

External evidence recommends the same caution. Reviews report strong evidence for effects of physical activity on mathematics performance and weaker evidence for other outcomes (44), small and heterogeneous effects of physically active lessons (45, 46), and a modest position for aerobic and resistance training among methods for improving executive functions (47). The quantity of movement is thus a weak predictor, and structure and cognitive demand are the likely moderators, which is precisely what the body percussion literature leaves undescribed.

### 3.3 The premise as a testable hypothesis: a protocol for surveying reporting practices

Sections 3.1 and 3.2 rest on a claim the paper does not demonstrate: that intervention studies in body percussion do not report the pedagogical variables their theory identifies as decisive. It is therefore stated as hypothesis H1: in the published corpus, the five items of the reporting standard of section 8 are absent from most reports, and pulse source in particular is specified in fewer than 20% of them. Labels beginning with H denote hypotheses about the field itself, testable against existing or observational material (H2 in section 4, H3 in section 5.3); labels beginning with P denote propositions about the empirical consequences of the framework, which require experimental manipulation (P1 to P10, section 7).

H1 requires no new data collection. Eligibility: peer-reviewed reports of body percussion interventions in any age group published from 2001 onwards, retrieved from Web of Science, Scopus, Dialnet and ERIC, with backward citation searching of the most recent bibliometric analysis (15). Coding: two independent raters, blind to authorship where feasible, apply Table 2 to the methods sections. Reliability: Cohen's kappa per item, with disagreements resolved by a third rater. Analysis: proportion of reports including each item, with 95% confidence intervals. The survey should be preregistered and the coding sheet deposited openly. It has not yet been conducted.

**Table 2:** Coding scheme for the proposed survey of reporting practices (H1).

Item	Question asked of the methods section	Coded present when
Pulse source	Who or what generated the beat during the intervention?	The text names the source (teacher, recording, metronome, participants) unambiguously
Tempo control	Could participants modify the tempo?	The text states whether tempo was fixed, negotiated or emergent
Error definition	How was incorrect performance defined?	The text specifies the criterion, e.g., deviation from model, loss of group coordination, or none
Teacher role	Did the instructor practise with the group or only direct it?	The text describes the instructor's participation during the activity
Assessment target	Did the instrument record product or process?	The text identifies the outcome measured and the moment of measurement

Items correspond one to one with the reporting standard of Section 8. Coding applies to methods sections only.

To show that the scheme can be applied, it was applied to one report with a full methods section, the trial described above (48). This is a worked example with a sample of one, not a test of H1; the coding sheet, with the basis for each decision, is given in Supplementary Table S1. One item is present (assessment target), two are partial (teacher role, and pulse source, which can be inferred from the recorded music for the choreographic arm but not for the experimental arm) and two are absent (tempo control and error definition). The scheme therefore discriminates: the item reported is the one the field already treats as obligatory, and the absent items are among those the theory identifies as decisive. The partial codes are informative too, because inferring a pulse source from a description of materials is the kind of reader inference a reporting standard exists to remove.

If H1 is supported, the reporting standard of section 8 becomes an evidence-based recommendation. If it is refuted, the mismatch is smaller than claimed and the contribution reduces to the propositions of section 7, which remain testable independently.

## 4 Why external coupling became the default

The prescriptive structure documented above was not introduced by body percussion and is not entailed by it. It belongs to the setting into which the practice was inserted, and tracing the origin of a default is the most direct way of showing that it is a default rather than a necessity. Four constraints are relevant, two belonging to the room and two to the practice. None is a pedagogical doctrine, which is why they count as frame factors (17) rather than beliefs that argument alone could correct.

The first two belong to the room. (i) Its architecture. Mass schooling in the nineteenth century had to manage dozens of bodies at once with one adult and scarce resources, and the response was material before it was doctrinal. The classroom is a historically invented device that organizes relations between teacher, pupil and knowledge by distributing bodies in space (49), and school desks were designed to fix posture and orient the gaze forward, largely on medical grounds (50, 51). Movement was not forbidden; its initiation passed to the adult. (ii) The regime under which movement returned. Movement was readmitted in quantity while initiative was withheld, through compulsory gymnastics and drill (52, 53). The Italian case is the most instructive: from the 1870s to the 1970s, primary schools practised gymnastics between the desks, a practice legitimized in hygienic terms but sustained by the lack of adequate spaces and equipment (54). Body percussion shares the property that made desk gymnastics viable for a century: it requires no instruments, no displacement and no additional floor area. A practice that prospers because it fits a room tends to inherit the constraints of that room, and one of them bears directly on the argument: where many bodies perform from fixed positions, someone has to supply the beat, and it will not be the bodies.

The other two belong to the practice. (iii) The settlement of an earlier dispute. The place of movement in music teaching was argued out once before, and both sides accepted external coupling. Stetson (55) held that rhythm perception is inseparable from muscular movement, a view shared by the pedagogy of Jaques-Dalcroze, in which the body executes what the ear must learn, while Giddings (56) held that rhythmic exercises belonged to physical education and proposed finger tapping as a minimal substitute executable from the seat. They disagreed about how much the body should move and which subject should own it; neither questioned the direction of coupling. Giddings replaced marching with finger tapping and the teacher still determined onset and offset; Dalcroze had pupils walk the metre but set it at the piano. (iv) The terms of transmission. Content does not determine function. According to the documentary reconstruction of (57), based on a previously unexamined collection in the Spanish General Archive of the Administration, the Orff-Schulwerk reached Spain from 1962, and the Sección Femenina, the women's section of the single party of the Franco regime, was among its most prominent agents, organizing training courses between 1964 and 1972. The authors conclude that its arrival was not fortuitous and examine the ideology associated with its dissemination. The data are theirs; the interpretation offered here is mine. What was disseminated was a repertoire of bodily activities whose purpose was set elsewhere: movement in abundance, with initiative retained. The four constraints are independent in origin and convergent in effect. None of them decided who holds the pulse; together they made the question unnecessary.

The historical account is an explanatory claim with a testable consequence, stated as H2: if external coupling is inherited from the school setting rather than entailed by body percussion, the same practice should show systematically higher CAI in schools than in the community contexts where it also occurs, such as drumming circles, samba and batucada groups and children's informal hand-clapping games. A default belonging to the method should travel with it; one belonging to the setting should not. H2 is operationalized as proposition P10. If CAI proves equally high in community settings, the historical explanation is superfluous.

## 5 Coupling architecture as a design variable

The preceding sections converge on one proposal: body percussion sessions should be characterized and reported by the coupling architecture they implement. Under external entrainment the beat comes from a source that does not adjust to the performers, and success is fidelity to it. Under mutual entrainment the beat is produced by the performers, and the relevant outcome is the stability and recoverability of collective coordination. Table 3 contrasts the two across nine dimensions. They are endpoints of a continuum, and most sessions combine them: a session may begin with an external source and shift to mutual coupling once a temporal scheme is stable, a sequence that corresponds to finding, keeping and being in phase with the beat (28). The claim is not that mutual coupling is always preferable, but that the combination is currently neither deliberate nor documented.

**Table 3:** External and mutual entrainment as coupling architectures in a body percussion session.

Dimension	External entrainment (unidirectional)	Mutual entrainment (bidirectional)
Source of the pulse	Teacher, metronome or recording	The participants' own body sounds
Direction of adaptation	Performers adapt to the source	All participants adapt to one another, or to the group aggregate (Section 5.1)
Tempo	Fixed; not modifiable by performers	Emergent; any participant can pull the group
Status of the demonstrated pattern	Reproduction target	Constraint and entry point for search
Definition of error	Deviation from the model	Loss of collective coordination
Function of variability	Noise to be reduced	Medium of adaptation
Teacher's body	Model to be copied	One coupled participant among others
Primary outcome measure	Fidelity, assessed after practice	Stability and recovery, assessed during practice
Synchronization signature	Adaptation to an unresponsive reference	Reciprocal inter-onset interval adjustment

The final row refers to the adaptation signatures of Konvalinka et al. (6). The mean-field regime is discussed in sections 2.2 and 5.1.

### 5.1 A continuous index rather than a dichotomy

No classroom session implements either architecture in pure form. Where a teacher holds the pulse, learners still influence one another through mutual audibility; where a group generates its own, leaders emerge. A continuous quantity is therefore proposed instead of a dichotomy: the Coupling Asymmetry Index (CAI), the degree to which influence over the group's timing runs in one direction only. CAI approaches 1 when one source determines the timing of all others and receives no influence in return, and 0 when influence is fully reciprocal. Predictions are stated as monotonic functions of CAI rather than as contrasts between conditions, which also accommodates change within a session: a session may begin near CAI = 1 and move lower as the group takes over the pulse.

The mean-field regime of Section 2.2 must be distinguished from all-to-all reciprocity, and the two can be separated empirically: under mean-field coupling, pairwise directed estimates are diffuse and weak while coupling to the ensemble aggregate remains strong, so individual-to-mean coupling should be estimated alongside the pairwise network. Two consequences follow. CAI remains low under mean-field coupling, because influence is not concentrated in any node, which is the correct verdict for the group. But the aggregate is itself unresponsive to any single participant as the ensemble grows, because one participant's influence on it tends to zero, so the architecture each member experiences approaches the external case although no external source is present. Mutual entrainment therefore does not scale indefinitely, and CAI should be reported with group size (section 5.4).

### 5.2 Measurement

Because the architectures differ in the direction of adaptation, CAI can be estimated from onset series with established procedures rather than judged by inspection. Four are proposed (Figure 1). (i) Cross-correlation of inter-onset intervals at lags zero and plus or minus one identifies reciprocal adjustment: bidirectional coupling produces the opposite-direction adjustment reported by Konvalinka et al. (6), whereas unidirectional coupling produces adaptation to a reference without a reciprocal signature. (ii) Phase- and period-correction estimates quantify how much each participant adjusts, and to whom (7, 8). (iii) Model-free directed-coupling estimates, such as Granger causality or transfer entropy, index the asymmetry of influence. (iv) Ensemble tempo drift is diagnostic in itself, because under mutual coupling group tempo is free to drift. Drift may also be directionally predictable: if preferred pulse rates are calibrated to the mechanics of the effectors that produce them, as has been argued for musical meter (43), a group released from an external reference should drift towards the natural frequencies of the body sounds it uses.

**Figure 1:**
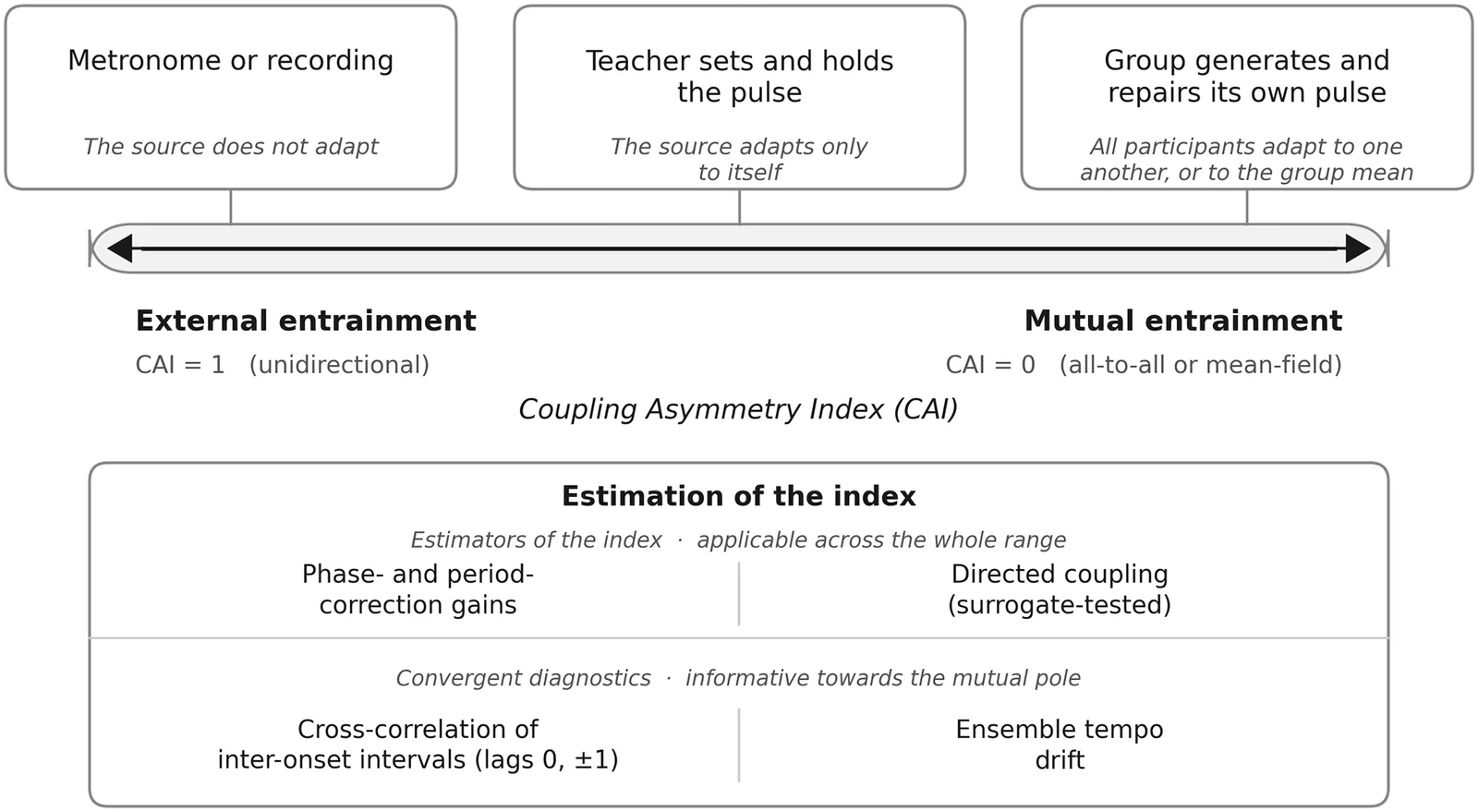
The coupling continuum and its estimation. CAI, Coupling Asymmetry Index. The three examples are illustrative positions, not discrete categories. In the lower band, the upper row lists the two procedures from which the index is estimated, applicable across the whole range; the lower row lists two convergent diagnostics whose informativeness is asymmetric, since the reciprocal adaptation signature and free tempo drift are both absent at the external pole by construction. All are described in Section 5.2.

Procedures (ii) and (iii) supply the directed estimates from which CAI is computed, the former where the weak-coupling assumption holds and the latter where it does not. Procedures (i) and (iv) are convergent diagnostics: the lag-one signature shows that bidirectional adaptation is present without apportioning it among participants, and drift requires no network estimation. Disagreement between estimator and diagnostics, such as a low CAI with no drift at all, indicates that the network has been misestimated. The diagnostics are informative in one direction only, since at the external pole the absence of drift and of a reciprocal signature follows by construction.

The formal definition is assembled from existing measures, because the quantity at issue, the asymmetry of directed influence among coupled rhythmic agents, has been formalized outside this literature. The pairwise gains are estimated as Wing et al. (58) estimated them for every pair of players in a string quartet, by fitting a first-order linear phase correction model to onset times. In its simplest form, for a group of *N* nodes,

$$t_{i,k+1}=t_{i,k}+T_{i}-\sum_{\;j\neq i}\alpha_{ij}(t_{i,k}-t_{\;j,k})+\varepsilon_{i,k}$$ (1)

where *t_i,k_* is the *k*th onset of participant *i*, *T_i_* the period of that participant's timekeeper, *ε_i,k_* a noise term, and *α_ij_* ≥ 0 the proportion of the asynchrony with *j* that *i* corrects on the next interval, that is, the influence of *j* on *i*. An external source, when present, enters as an additional node *s* whose own gains *α_sj_* are zero by construction, so that *N* = *n* + 1 for *n* participants. Negative estimates, and gains that do not exceed the distribution obtained from phase-randomized surrogates, are set to zero. For each pair, the directionality index of Rosenblum and Pikovsky (59), which indicates whether coupling is unidirectional and how asymmetric it is otherwise, is then

$$d_{ij}=\frac{\alpha_{\;ji}-\alpha_{ij}}{\alpha_{ij}+\alpha_{\;ji}}$$ (2)

which equals +1 when *i* drives *j* without being driven in return, −1 in the reverse case and 0 under perfect reciprocity. CAI aggregates these indices over the *N*(*N*−1)/2 pairs, weighting each by its total coupling strength *w_ij_* = *α_ij_* + *α_ji_*:

$$CAI=\frac{\sum_{i<j}|\alpha_{ij}-\alpha_{\;ji}|}{\sum_{i<j}(\alpha_{ij}+\alpha_{\;ji})}=\frac{\sum_{i<jwij}|d_{ij}|}{\sum_{i<jwij}}$$ (3)

Because |*a*−*b*| = *a* + *b*−2 min(*a*, *b*), Equation 3 can be written equivalently as

$$\mathrm{CAI}=1\text{-}r,\;r=\frac{\sum_{i\neq j}\min(\alpha_{ij},\alpha_{\;ji})}{\sum_{i\neq j\alpha ij}}$$ (4)

where *r* is the weighted reciprocity of Squartini et al. (60), so that no new statistic is introduced. CAI is the proportion of the group's total corrective adjustment that is not returned. It is 0 when every coupling is matched by an equal coupling in the opposite direction, 1 when no coupling is returned, and undefined only when no coupling is present. Where model-free estimators are used, their pairwise directed strengths replace the gains in Equations 2–4.

Weighting, rather than a simple mean of |*d_ij_*| over pairs, is what makes the index invariant to group size. A simple mean counts every pair, and the pairs among participants grow as *n*(*n*−1)/2 whereas the pairs linking participants to an external source grow only as *n*. For a source that drives *n* participants who also adjust reciprocally to one another, the unweighted mean therefore equals 2/(*n* + 1), falling from 0.67 with two participants to 0.08 with twenty-five although the architecture is unchanged. Under Equation 3, suppose each participant allocates a total correction *γ*, a share *α* to the source and the remainder equally among the other participants: the numerator is *nα*, the denominator *nγ*, and CAI = *α*/*γ* for any *n*. A source that alone drives the group gives CAI = 1, and all-to-all or mean-field reciprocity gives CAI = 0, at every group size. The dependence that Equation 3 retains belongs to the architecture, not to the estimator: if each pairwise coupling keeps its strength as the group grows, total peer influence grows with *n* and CAI falls. Weighting also ensures that pairs with near-zero gains, whose directionality ratio is dominated by estimation noise, contribute in proportion to their strength.

Two further properties should be reported with CAI. The first is hierarchy. A strong but responsive leader emits a large share of the influence while also receiving it, so a group can be hierarchical and reciprocal at once; the global reaching centrality of Mones et al. (61) quantifies the hierarchy of the estimated network. The second is chance reciprocity: in a dense network some reciprocation is expected whatever the architecture, so *r*, and with it CAI, should also be reported relative to its expectation under a null model that preserves each participant's total incoming and outgoing gain, as Squartini et al. (60) do for weighted networks, extending the correction of Garlaschelli and Loffredo (62) for binary ones. Because coupling functions can be inferred as they evolve (63), Equations 1–4 can also be applied in successive windows, which the claim of Section 5.1 that a session may shift from external to mutual coupling requires.

Three cautions apply. First, directed-coupling estimators such as Granger causality can give spurious results on strongly periodic series, so onset series should be reduced to inter-onset intervals or phase residuals and tested against phase-randomized surrogates. Estimators less exposed to this problem include the phase-slope index, which is insensitive to mixtures of independent sources (64), and convergent cross mapping for deterministic nonlinear systems (65). Second, where weak coupling is tenable, phase-based estimators derived from coupled-oscillator models are preferable to purely statistical ones: their parameters are interpretable as the proportion of an asynchrony corrected on the next interval and comparable with the sensorimotor synchronization literature (7, 66, 67), and phase reduction makes the coupling function identifiable for weakly coupled limit-cycle oscillators (26, 68, 69). Model-free measures are also sensitive to preprocessing: Granger causality is invariant under invertible filtering, so band-pass filtering cannot isolate frequency-specific influence and degrades estimation (70). Where coupling is strong or markedly non-sinusoidal, model-free measures are more robust; Granger causality applied to body sway has recovered the leadership structure of string quartets (71). Third, in larger groups the network may fragment, so community detection should be reported with the global index, since an intermediate CAI may reflect two internally mutual subgroups.

### 5.3 Proposed mechanism

The mechanism is stated as hypothesis H3 in four links, so that each proposition can be referred to the step it tests. M1: bidirectional coupling makes the timing target contingent on the learner's own output, because each participant adjusts to partners who are adjusting in turn, whereas an unresponsive source allows convergence on a stable solution (6, 8). M2: a contingent target prevents early stabilization and sustains exploration. M3: sustained exploration expands the repertoire of coordinative structures that achieve the same rhythmic function, the property described as degeneracy (29). M4: a degenerate repertoire supports transfer, because novel tempi and patterns can be met by recruiting an available alternative rather than by relearning.

The operative property is contingency, not variability, and this is what makes the framework falsifiable. An irregular but unresponsive source also presents a moving target. Under such a source, however, a participant's correction leaves the target unchanged, so one adequate tracking solution remains available; under mutual coupling, each participant's adjustment changes the problem for the others. The external-irregular condition of Section 7 is therefore the principal test, and the framework predicts that variability without contingency does not suffice. The same contrast separates the proposal from two established routes to exploration. High contextual interference varies the practice schedule and the constraints-led approach varies task constraints (18, 19); in both, the variation is arranged in advance and does not depend on what any learner does. All three accounts predict better transfer (P2), so P2 alone cannot show that this account adds anything. They diverge in the external-irregular condition, where an interference account expects a benefit and this one does not.

The links attach to different propositions. M1 alone suffices for faster recovery, since a group in which every member corrects towards every other re-converges without any enlarged repertoire; P3 tests this. P1 tests M2, and only P2 requires all four links. M3 is the weak point, because exploration during practice and transfer afterwards bracket the repertoire without measuring it: a failure of P2 with P1 intact could not distinguish a repertoire that never formed from one that did not transfer. The measure for P1 therefore includes a delayed count of distinct solutions produced with no model present. The chain also predicts a cost: continuous correction should depress performance on the trained pattern during acquisition, as high contextual interference does (30, 31).

Claims that body percussion develops fundamental movement skills are transfer claims, whether or not they are stated as such. On this account what transfers is an enlarged repertoire of coordinative solutions rather than an abstract representation, so transfer should appear on tasks that afford alternative solutions and not on tasks that require a single prescribed pattern. A repertoire account also predicts transfer by interpolation within the range of solutions explored but not by extrapolation beyond it, whereas an abstraction account predicts both; P2 therefore tests tempi and patterns inside and outside the explored range separately. Two restrictions on generality follow. The transfer at issue is across rhythmic patterns and tempi, not across task domains. And because repertoires are enlarged in individuals while coupling architecture is a property of the group, P1 and P2 are measured on individuals within groups, with the group as the unit of randomization.

M1 implies that what matters is the cognitive demand of the coordination rather than the amount of movement. A related claim underlies the trial described in Section 3.2: repetitive automatized movement places low demands on executive control, whereas dual tasking requires continuous engagement. That account is a rival specification of the active ingredient. On it the operative variable is the number of concurrent task streams; on this one it is the direction of the timing information. The two are separable, because load can be raised by pattern complexity while the pulse remains external, but as Table 1 shows, in the published comparisons the low-load conditions are also the high-asymmetry ones, so every existing contrast varies both at once. This is not a criticism of studies that were not designed to address the question. The factorial test of proposition P4 would discriminate the two accounts.

### 5.4 Boundary conditions

The framework does not claim that lower CAI is always preferable, and four boundary conditions limit it. (i) Initial acquisition: when learners have no temporal scheme, an external and regular source is likely to be superior, which corresponds to the finding-the-beat phase of Moens and Leman (28) and to the role that Dreyfus and Dreyfus (24) attribute to instruction. (ii) Group size and spatial dispersion: mutual coupling requires mutual audibility, and beyond some number of participants, or once performers move through space as locomotor work requires, reciprocity between identifiable individuals cannot be sustained. The influence structure may then fragment into internally mutual subgroups or converge on the mean field, two outcomes that the procedures of Section 5.2 distinguish. The thresholds are empirical questions, and the condition matters particularly for gross motor content. (iii) Age and prior training: young children synchronize better with a responsive partner than with a machine (25), which favours mutual coupling, but repairing a collective pulse requires timing control established first. (iv) Task goal: where the objective is a fixed ensemble performance reproduced with an external reference, high CAI is a requirement. These conditions predict an interaction rather than a main effect: the advantage of low CAI should increase with prior competence, decrease with group size and be absent at the outset of learning (P8).

### 5.5 coupling asymmetry and autonomy support

The proposal that learners should hold some control over practice conditions connects with a contested literature. The OPTIMAL theory holds that autonomy support facilitates motor learning through motivational and attentional pathways (72), and studies of self-controlled practice report advantages of learner-controlled over yoked schedules (73). A meta-analysis, however, found this literature underpowered and biased, and an exploratory random-effects estimate from four preregistered experiments was g = 0.02, 95% CI [−0.17, 0.21] (74).

CAI is not a new name for autonomy support. Autonomy support concerns the choices a teacher grants, over repertoire, order, partners or feedback. CAI concerns the temporal structure of the task: from whom the timing information flows, and whether it flows back. The two are orthogonal. A teacher may grant extensive choice over repertoire, order and grouping while keeping the pulse at the piano, which combines high autonomy support with CAI near 1. A directive teacher may set all three and then require the group to establish and repair its own pulse, which combines low autonomy support with low CAI. Because all four combinations are realizable, the constructs can be dissociated experimentally (P9).

The distinction also has a theoretical consequence. Autonomy support is held to operate motivationally. The mechanism of Section 5.3 is not motivational: it runs through the contingency of the timing target, exploration and degeneracy, and predicts effects on coordination variables that a motivational account does not. If CAI effects appeared only on affective measures and not on variability, transfer or recovery, the framework would be wrong and the phenomenon would belong to the autonomy literature.

## 6 An observational framework for applied settings

Instrumented measurement will be unavailable in most classrooms. Three questions approximate the same variable at coarser resolution: who initiates the movement, whether it requires permission, and whether it counts towards assessment (Table 4). They are ordered by increasing difficulty of change: the first can be altered within a session, the second requires changing classroom norms and the third the curriculum. They correspond to the functions of education distinguished by Biesta (12), which is a reason for selecting them, not an empirical claim.

**Table 4:** Observational indicators for the three questions.

Question	High-asymmetry indicator (CAI towards 1)	Low-asymmetry indicator (CAI towards 0)	Proposed record
Who initiates the movement?	Teacher sets and holds the tempo; learners enter on cue	Learners propose tempo; entries are negotiated	Proportion of session time in which the pulse source is a learner
Does it require permission?	Learners await authorization to stand, move or vary	Learners modify position, dynamics or pattern unprompted	Rate of permission requests and of unprompted variations per 10 min
Does it count towards assessment?	Assessment records reproduction of the pattern	Assessment records adaptation, recovery and contribution to coordination	Presence and weight of process criteria in the rubric

Indicators are proposed here for the first time and require validation, including inter-rater reliability and comparison against Section 5.2. The column labels refer to coupling asymmetry, not autonomy support, which Section 5.5 treats as orthogonal: only the first is a direct, if coarse, proxy for coupling architecture; the others capture conditions that covary with it.

The relation between these indicators and CAI is assumed, not established, and must be tested before they are used as a proxy: recorded sessions spanning the full range of arrangements should be scored with Table 4 while onset series are collected in parallel. Scored so that higher values indicate learner-distributed influence, the composite should correlate negatively with CAI, and a coefficient of about −0.5 to −0.7, with an intraclass correlation of at least 0.75 for each indicator, would suffice for a coarse proxy. The indicators should not behave alike. The initiative indicator should carry most of the association, being the only one defined by the timing structure itself; the permission and assessment indicators describe institutional conditions that covary with coupling architecture and, on the argument of Section 5.5, may correlate more strongly with autonomy support. A composite whose association is carried by those two items would measure classroom climate rather than coupling architecture; the indicators would then remain useful to teachers but could not be reported as a proxy.

## 7 Testable propositions

The propositions in Table 5 state the empirical content of the argument, each with a prediction, a measure and a disconfirming result. P1 to P3 test the three consequences of the core claim. P4 and P9 test it against the accounts that would make it redundant, cognitive load and autonomy support. P5 concerns affective effects that should accompany the shift without carrying it. P6 and P7 test two pedagogical levers, the participating teacher and process criteria in assessment, with CAI as mediator. P8 states the moderation implied by Section 5.4, and P10 operationalizes H2. Three design requirements apply throughout.

**Table 5:** The ten propositions, with predicted outcomes, measures and disconfirming results.

Proposition	Prediction	Measure	Disconfirmation
P1 Exploration	Movement variability and number of distinct solutions increase as CAI decreases, and the delayed repertoire count increases with them	Kinematic variability from inertial sensors; count of distinct movement solutions during practice, and a delayed count produced with no model present, which indexes the repertoire itself (M3)	No monotonic relation with CAI, or variability equally high in the external-irregular condition
P2 Transfer	Lower CAI yields better delayed transfer to untrained patterns and tempi, even if fidelity on the trained pattern is equal or lower	Delayed transfer tests at novel tempi and patterns inside and outside the range explored during practice, analysed separately; acquisition curve reported separately	No transfer advantage; advantage equally present in the external-irregular condition; or equal transfer inside and outside the explored range, which favours an abstraction account over the repertoire account of Section 5.3
P3 Recovery	Groups trained at low CAI re-establish coordination faster after perturbation	Time to re-synchronization and asynchrony variance after a tempo step or performer removal	Equal or slower recovery at low CAI
P4 Discrimination from cognitive load	Crossing concurrent load (single vs. dual task) with CAI yields a crossed dissociation: both factors affect acquisition, CAI in the costly direction predicted in Section 5.3, while only CAI affects variability, transfer and recovery	Two-by-two factorial design with the measures above	Any failure of the dissociation: effects of CAI disappear once load is held constant; load affects transfer or recovery; or the two factors interact. The first outcome leaves the framework redundant rather than false
P5 Affect and persistence	Lower CAI increases perceived autonomy and reduces state anxiety, and perceived autonomy mediates persistence in voluntary practice	Validated autonomy and state-anxiety scales after each session; voluntary practice logged over the following weeks; mediation model with group as random effect	No difference in perceived autonomy across CAI levels, or an effect on autonomy that does not mediate persistence
P6 The participating teacher	A teacher who practises with the group and whose failures are visible elicits more exploratory behaviour than a teacher who only demonstrates, at equivalent CAI	Rate of unprompted variation and number of distinct solutions as dependent variables; CAI as manipulation check; instructional content held constant across teacher conditions	No difference in exploratory behaviour once CAI is held constant
P7 Assessment washback	Introducing process criteria into assessment lowers CAI, and raises exploratory behaviour through that change rather than independently of it, more than increasing movement time does	Factorial design crossing assessment criteria (product vs. process) with session duration; CAI estimated from onset series as mediator; exploratory behaviour coded from video	Movement time produces effects equal to or larger than those of assessment criteria; or exploratory behaviour changes with no accompanying change in CAI, which would show that the lever does not operate through coupling architecture
P8 Moderation	The advantage of low CAI increases with prior timing competence and decreases with group size, and is absent or reversed during initial acquisition; beyond the size at which coupling becomes mean-field, a low estimated CAI should no longer predict the outcomes of P1 to P3	Competence assessed at baseline with a synchronization task; group size manipulated between conditions, with individual-to-mean coupling estimated alongside the pairwise network so that fragmentation into subgroups can be distinguished from convergence on the mean field; acquisition phase analysed separately	The effect of CAI is constant across competence levels, group sizes and phases of learning
P9 Discriminant validity	In a design crossing autonomy support with CAI, coupling architecture affects coordination variables independently of autonomy support, and autonomy support affects motivational variables independently of CAI	Autonomy support manipulated as choice over repertoire, order and grouping; coordination and motivational outcomes measured separately	The two manipulations produce the same pattern of effects, indicating that CAI is a redundant relabelling of autonomy support
P10 Context	Estimated CAI is systematically higher in school implementations than in community implementations of comparable rhythmic practices	Same estimation procedure applied in both settings to matched repertoires; drumming circles, samba and batucada groups and informal hand-clapping repertoires as community comparisons	The two contexts do not differ, which would remove the explanatory need for the historical account of Section 4

Propositions are grouped by function as set out in the text. P4 is the most informative experiment proposed, because it sets the framework against the account currently dominant in the field.

### 7.1 Isolating the variable

Conditions must be matched for time on task, material complexity and instructional content, and two conditions do not suffice: generating and repairing a shared pulse is harder than tracking a metronome, so an effect could be attributed to difficulty. A third condition is required in which the external source is irregular but unresponsive, with timing fluctuations matched to those a human group produces, so that difficulty and unpredictability are equated while contingency is withheld (Section 5.3).

### 7.2 Measuring the right things at the right level

CAI serves as the manipulation check, and behavioural measures such as the rate of unprompted variation serve as dependent variables. Outcomes must be assessed under conditions common to all groups, since testing each group under its own architecture would make faster recovery a consequence of the definition rather than a finding. The group is the unit of analysis, so analyses must be multilevel and report intraclass correlations; power depends on the number of ensembles rather than of children, and initial tests are best run with groups of three to five, where pairwise reciprocity can be distinguished from the mean field. Effect sizes are not stipulated, since the framework predicts direction and pattern rather than magnitude. The propositions also address different constructs from the corpus they criticize: published trials report executive function, attention and socio-emotional outcomes, whereas the propositions concern coordination variability, transfer and recovery. Linking the two would require the path from coupling architecture through coordination to cognition to be measured in full.

### 7.3 Controlling the obvious rivals

Perceived enjoyment, novelty and social connectedness should be measured as covariates, since producing one's own pulse may simply be more engaging; the framework predicts that effects on coordination variables survive their inclusion. Physiological intensity should also be recorded, because it may differ between conditions. Preregistration, active controls and delayed retention and transfer tests are assumed.

P2 is risky in the relevant sense: it allows prescriptive practice to outperform mutual coupling on the trained pattern while losing on transfer, so the framework accepts contradiction by the measure the field reports most often. Four outcomes would falsify it. If lowering CAI does not increase exploratory variability, M1 or M2 is broken. If P1 holds but P2 fails and the repertoire count is unchanged, M3 has failed; if the repertoire is enlarged and transfer is not, M4 has. If the predicted effects appear equally in the external-irregular condition, the operative property is variability rather than contingency. Each of these outcomes requires abandoning the mechanism as stated. Two further outcomes would leave the framework not false but redundant: no effect of CAI once cognitive load is held constant (P4), or effects of CAI indistinguishable from those of autonomy support (P9). In either case the rival account would be preferable on grounds of economy.

## 8 Reporting standard

If coupling architecture is a variable, it must be reported. Five items would take fewer than a hundred words in a methods section and would make intervention studies comparable on this dimension: (1) pulse source, who or what generated the beat; (2) tempo control, whether participants could modify the tempo and whether it was fixed, negotiated or emergent; (3) error definition, whether solutions differing from the demonstrated pattern were accepted and on what criterion performance was judged incorrect; (4) teacher role, whether the instructor participated or only directed; and (5) assessment target, whether the instrument recorded product or process. A sixth item, the proportion of session time devoted to imitation rather than exploration, is worth reporting where records permit, but is not among the items coded in Table 2.

Beyond reporting, the field would benefit from design features that the bibliometric evidence cited in Section 1 shows to be uncommon: preregistration, active controls matched for engagement and cognitive demand, process measures alongside outcomes, delayed transfer tests, and descriptions detailed enough for replication. None of these is new in the movement sciences, but all are rare in this literature.

## 9 Implications for practice and teacher education

The framework does not recommend abandoning demonstration. It proposes that coupling architecture be chosen deliberately and varied within a session: external coupling to establish a temporal scheme, mutual coupling once the scheme is stable, and episodes in which the group repairs its own pulse unaided. Table 4 allows a teacher to check what was implemented rather than what was intended.

Because the default is a frame factor originating in the material organization of the classroom (Section 4), persuasion alone will not change it. The levers are those of propositions P7 and P8: the assessment system and the size of the working group. Teachers who are told that their practice is prescriptive without being given those levers are held responsible for a constraint they do not control. The same caution applies to learners: practices that require exploration presuppose space, materials and preparation that are unevenly distributed, and the position of learners with disabilities within such regimes remains largely unexamined (75). Conversely, the property that lets body percussion fit any room, that it needs no instruments, has been proposed as the basis of a guaranteed curricular minimum of rhythmic, ensemble-based and improvisatory participation achievable with the body and voice alone (42). The present analysis adds a condition: such a minimum would guarantee participation in making music, and not only in reproducing it, if it also specified who holds the pulse.

In teacher education the difficulty is structural, since a course about embodied pedagogy delivered through lectures reproduces the problem it describes. Van der Kamp et al. (16) describe a course in which students practise a self-chosen skill for a few minutes daily over five weeks while the instructor also practises and shares his progress, and report that students rated this practical below the lectures. Such preferences are themselves a product of prior schooling and should be treated as a variable rather than as an objection.

### 9.1 Feasibility and ethical considerations

Three practical obstacles should be acknowledged. Onset detection in a classroom is difficult, because several performers produce overlapping sounds of similar timbre; contact microphones or wrist and ankle sensors are more robust. Instrumentation alters the situation it measures, so habituation should precede data collection. And the estimation procedures of Section 5.2 require long uninterrupted passages, which constrains task design.

Research of this kind involves minors and requires guardian consent, participant assent, ethics committee approval, secure handling of video data and the option of participating without being recorded. Because the manipulation concerns the structure of ordinary lessons rather than a risk factor, the main ethical burden is data protection.

## 10 Limitations

This is a conceptual contribution and reports no new data. The propositions of Section 7 are untested, and some are likely to fail. The characterization of current practice rests on published descriptions, bibliometric evidence and the author's experience rather than systematic observation; producing that evidence, with Table 4 validated against the procedures of Section 5.2, is the next step. The historical material is European and predominantly Spanish. The index has not been computed on real data: its invariance to group size is shown analytically in Section 5.2, but its behaviour with small ensembles, noisy onset detection and fragmented networks is unknown. Finally, the author developed one of the methods discussed, and the analysis is critical of a literature to which he has contributed (see Conflict of interest).

## 11 Conclusion

Body percussion entered classrooms with an embodied justification and a prescriptive practice. The mismatch reflects inheritance rather than inconsistency: the school setting had already settled who initiates movement, in favour of the adult and for logistical reasons. Desk gymnastics survived for a century in Italian schools because it fitted the room, and body percussion is popular today partly for the same reason.

Body percussion is also the practice best placed to escape that inheritance, because the sound source is the body and there are as many sources as participants. Coupling architecture is therefore a free design variable, and one that established measures can estimate. The question this paper places on the research agenda is not whether learners move, or how much, but how influence over the shared pulse is distributed and what that distribution changes. A field that explains learning through emergence should be able to report, for each intervention, whether anything was allowed to emerge.
