# Supplementary material for: Teaching the moving body with a still pedagogy: body percussion and the coupling architecture of embodied learning in physical education
Source: Front Sports Act Living. 2026 Oct 14;8:1963569. doi: 10.3389/fspor.2026.1963569 (PMC13714971; doi:10.3389/fspor.2026.1963569)
Supplement: Supplementary file 1 [file Table1.docx]

**Supplementary Material**

*Teaching the Moving Body with a Still Pedagogy: Body Percussion and the Coupling Architecture of Embodied Learning in Physical Education*

**Francisco Javier Romero-Naranjo^1*^**

*^1^Department of Innovation and Didactic Training, University of Alicante, Alicante, Spain*

*** Correspondence:**
Francisco Javier Romero-Naranjo
bapne.central@gmail.com

**1**	**Coding sheet for the worked example of section 3.3**

Section 3.3 of the main text reports the application of the five-item coding scheme to one report for which a full methods section is available, the randomized controlled trial of Romero-Naranjo and Navarro-Camacho (2026a). The complete coding sheet is reproduced below. This is a worked example with a sample of one, not a test of hypothesis H1. It is included to show that the scheme can be applied to published reports and discriminates between items, and to make each coding decision available for scrutiny.

Coding rule applied: an item is coded present when the methods section states the information explicitly; partially present when it can be inferred with reasonable confidence from the description of materials or procedures but is not stated; and absent when neither is the case. The third column paraphrases the relevant passage rather than quoting it; in the full survey, recording the supporting passage verbatim would allow each decision to be audited.

**TABLE S1 |** Coding sheet applied to Romero-Naranjo and Navarro-Camacho (2026a).

| **Item** | **Code** | **Basis for the decision** |
| --- | --- | --- |
| Pulse source | Partially present | For the choreographic arm, sequences are described as performed to recorded pop, rock and classical music, from which an external and unmodifiable pulse can be inferred. For the experimental arm, the protocol is described in terms of dual tasking, dissociation and randomized instruction, without identifying what produced the beat. For the physical education and Orff arms, no pulse source is specified. |
| Tempo control | Absent | No statement in any arm as to whether participants could alter the tempo, or whether tempo was fixed by the instructor, by a recording or by the group. |
| Error definition | Absent | No criterion is given for what counted as incorrect performance during the intervention. Outcome instruments are described, but performance criteria within sessions are not. |
| Teacher role | Partially present | Sessions in the experimental arm are described as led by a certified trainer, which establishes direction but not participation; whether the instructor practised alongside participants is not stated. For the control arms the instructor's role is not described. |
| Assessment target | Present | The BRIEF-2 and the BOT-2 are identified as outcome instruments and were administered before and after the intervention, which establishes unambiguously that product rather than process was recorded. |

*Note.* Codes were assigned by a single coder; no inter-rater reliability is available for a sample of one. The survey specified in section 3.3 requires two independent coders and the reporting of Cohen's kappa per item.

One feature of this coding matters for replication. The partial codes arise from reader inference: attributing a pulse source to an arm on the basis of the musical materials used is the inference a reporting standard exists to make unnecessary, and a coder working from a different reading of the same materials could reasonably have recorded those items as absent. Stating the coding rule explicitly allows such disagreements to be identified and resolved.

**References**

Romero-Naranjo, F. J., and Navarro-Camacho, V. R. (2026a). Neuromotricity and ADHD in primary school students: a randomized controlled trial. *Int. Res. J. Multidiscip. Scope* 7, 881–891. doi: 10.47857/irjms.2026.v07i03.010753
